# Morphological Differentiation May Mediate Mate-Choice between Incipient Species of *Anopheles gambiae* s.s.

**DOI:** 10.1371/journal.pone.0027920

**Published:** 2011-11-21

**Authors:** Michelle R. Sanford, Berna Demirci, Clare D. Marsden, Yoosook Lee, Anthony J. Cornel, Gregory C. Lanzaro

**Affiliations:** 1 Department of Pathology, Microbiology, and Immunology, School of Veterinary Medicine, University of California Davis, Davis, California, United States of America; 2 Faculty of Science, Hacettepe University, Ankara, Turkey; 3 Department of Entomology, University of California Davis, Davis, California, United States of America; Global Viral Forecasting Initiative, United States of America

## Abstract

The M and S molecular forms of *Anopheles gambiae* s.s. have been considered incipient species for more than ten years, yet the mechanism underlying assortative mating of these incipient species has remained elusive. The discovery of the importance of harmonic convergence of wing beat frequency in mosquito mating and its relation to wing size have laid the foundation for exploring phenotypic divergence in wing size of wild populations of the two forms. In this study, wings from field collected mosquitoes were measured for wing length and wing width from two parts of the sympatric distribution, which differ with respect to the strength of assortative mating. In Mali, where assortative mating is strong, as evidenced by low rates of hybridization, mean wing lengths and wing widths were significantly larger than those from Guinea-Bissau. In addition, mean wing widths in Mali were significantly different between molecular forms. In Guinea-Bissau, assortative mating appears comparatively reduced and wing lengths and widths did not differ significantly between molecular forms. The data presented in this study support the hypothesis that wing beat frequency may mediate assortative mating in the incipient species of *A. gambiae* and represent the first documentation of a morphological difference between the M and S molecular forms.

## Introduction

As one of the most important vectors of *Plasmodium falciparum* in west Africa, *Anopheles gambiae* s.s. has been the subject of great interest, with respect to speciation, population structure and gene flow. *A. gambiae* s.s. is one member of the six species *A. gambiae* complex which are considered morphologically indistinguishable [1] and is also a developing model of speciation in sympatry [2], [3], [4], [5]. The two molecular forms: M and S, identified by a single nucleotide polymorphism in the ribosomal intergenic spacer [6], [7], have been shown to have phenotypic divergence in different locations within their geographic range [8] that has led to their designation as incipient species. The most notable phenotypic differences documented between the forms, thus far, include differential insecticide resistance [9], desiccation resistance [10] and larval habitat segregation [11], [12]. It has been proposed that the mechanism responsible for promoting divergence is pre-zygotic [13] and associated with mate selection either during swarm formation [14] or within a swarm [15].

One of the most difficult aspects to assess in these incipient species has been the mechanism responsible for assortative mating in wild populations. Specifically, what phenotypic information do potential mates use to discriminate between “incipient conspecifics”. Recently acoustic information through the process of harmonic convergence has been suggested as a widespread mechanism for mate selection in mosquitoes [16].

The first evidence that harmonic convergence between male and female *A. gambiae* of the same molecular form came from work by Pennetier et al. [17]. Male and female mosquitoes of the M or S form were statistically more likely to harmonize wing beat frequency with individuals of the same molecular form as themselves. This suggests that, at least for close range interactions wing beat frequency through harmonic convergence [18] may provide the phenotypic information required for mate selection. Previous attempts to document a difference in wing beat frequency of isolated mosquitoes from the two forms did not yield a significant difference [19] and this is most likely due to the need of the mosquitoes to be in close proximity and to have the potential to harmonize wing beat frequencies. Further evidence for wing beat frequency convergence has come from the correlation between wing size, wing beat frequency and mate selection [20]. Cator et al. [20] showed that larger females, which are capable of carrying larger egg complements, have larger wings and higher wing beat frequencies than smaller females. They also demonstrated that wing beat frequencies were assessed by potential mates through harmonic convergence. These data suggest that wing beat frequency convergence confers information about an individual mosquito's fitness and local adaptation to a potential mate. Thus, it follows that wing size may be a measurable phenotypic character which can be used to analyze wild *A. gambiae* populations.

One other significant aspect of assortative mating in these incipient species is that different populations of *A. gambiae* s.s. exhibit different levels of reproductive isolation at different geographic locations across their range [4], [21]. In Mali where genetic divergence between molecular forms has been extensively studied [5], [22], [23], reported hybridization rates between the M and S forms are low [4] suggesting very strong assortative mating. In other parts of the sympatric distribution, such as in Guinea-Bissau, hybridization rates are much higher [24], suggesting assortative mating between molecular forms is reduced from the pattern associated with the area where the molecular forms were first described.

In this study, the length and width of female *A. gambiae* wings of both the M and S molecular forms were measured from two different locations in West Africa to examine phenotypic differences. Two predictions follow from the assumption that wing size, and hence wing beat frequency, confers sufficient phenotypic trait information for the differential assortative mating observed in wild populations. The first of these is that if wing size confers phenotypic information about mate quality and the local adaptation of an individual to a habitat, there may be a difference in wing size between countries attributable to local adaptation. The second prediction we can make is that where assortative mating is occurring, for example in Mali, there should be evidence of wing size differentiation between the molecular forms. The data show evidence of morphological differentiation at both the geographic and molecular form levels and support the hypothesis that wing beat frequency confers information critical to assortative mating in this species.

## Results

Mean wing size differed by both country and molecular form. Wing size, as measured by wing width, appears to be a measurable phenotypic trait that may differentiate the molecular forms of *A. gambiae* s.s. in certain parts of its distribution.

### Wing size by country

**Figure 1:**
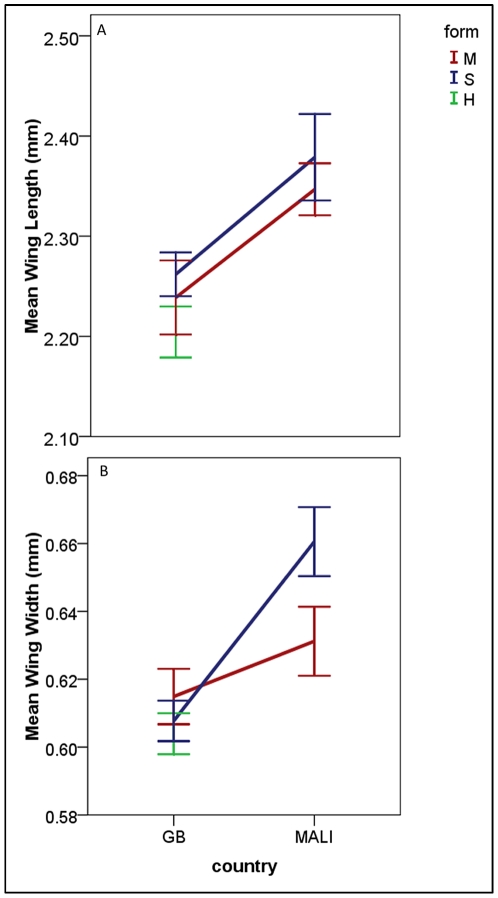
Mean wing size measurements of *Anopheles gambiae* from Guinea-Bissau and Mali. Mean wing length (A) and mean wing width (B) (+/− SEM) of right wings from female *Anopheles gambiae* s.s. collected in Guinea-Bissau (GB) and Mali by molecular form; Red – M molecular form, Blue – S molecular form and Green-hybrid form (a single hybrid collected in Mali is not included in this figure). The hybrid specimens were not included in the ANOVA but are displayed here for illustrative purposes.

Analysis of variance (ANOVA) for both mean wing length and mean wing width indicated a significant difference between Mali and Guinea-Bissau (Table 1). We observed that there was a significant difference in length (Figure 1A) and width (Figure 1B) of the wings between Guinea-Bissau and Mali. Guinea-Bissau mosquito wings were significantly smaller than those from Mali, regardless of molecular form. The potential for a site effect within each country was evaluated but failed to reveal significant differences (Supporting Information S1) and was dropped from the overall ANOVA.

**Table 1:** Analysis of variance tables for the analysis of wing length and wing width for female *Anopheles gambiae* s.s. right wings collected in Mali and Guinea-Bissau for the factors: country of origin for the mosquito specimen, molecular form (M or S), and the interaction of these factors.

Factor	Mean Square	d.f.	F	p-value
**Wing Length**				
**Country**	2331.398	1	10.654	0.002*
**Form**	140.531	1	0.642	0.425
**Form×Country**	3.589	1	0.016	0.898
**Error**	218.821	92		
**Wing Width**				
**Country**	271.825	1	15.091	<0.001*
**Form**	27.990	1	1.554	0.215
**Form×Country**	75.942	1	4.216	0.042*
**Error**	18.012	108		

*indicates a significant difference at the α = 0.05 level.

### Wing size by molecular form

The significant difference observed in wing size between the M and S molecular forms was dependent on the aspect of wing size evaluated. Mean wing lengths were not significantly different between forms (Table 1; Fig. 1A) however; mean wing widths were significantly different in Mali (Table 1; Fig. 1B). This is the first documentation of a morphological difference between the M and S molecular forms.

Hybrid specimens did not present an intermediate phenotype. A single hybrid specimen was collected in Mali and was removed from further analysis (Supporting Information S1). Hybrid specimens from Guinea-Bissau appeared smaller in both wing length and wing width measurements (Fig. 1A–B). Mean wing length of hybrids was more similar to the mean wing length of M form mosquitoes while mean wind widths were more similar to those of the S form consistent with recent genetic analyses [25], although these trends were not statistically significant (Supporting Information S1).

## Discussion

### Morphological Differentiation

These data comprise the first documentation of a morphological difference between the M and S molecular forms of *A. gambiae* s.s. This is an important finding for several reasons. Although the molecular forms have been considered incipient species for some time [6] the extent to which they are genetically divergent and reproductively isolated has been debated [3], [4], [23], [24], [26]. The search for phenotypic differences has included a range of different behavioral, phenological and physiological differences (for a review see: [8]) which has provided many different avenues to pursue and lots of variation. However, few traits allow for easy rapid measurement on a large scale. Although the data presented here are too limited in geographic distribution and sample size to be used as a discriminating factor to replace the use of a PCR diagnostic in these species [27], [28], they may provide a useful addition to existing data collection that can be rapidly assessed on a large scale.

The pattern of hybridization between the M and S forms in Guinea-Bissau is not only higher than in other locations but is characterized by backcrossing of individuals [21], [24], [25]. The data for both wing length and wing width of hybrids collected in Guinea-Bissau, in this study, did not show an intermediate phenotype as might be expected for F_1_ hybrid individuals. If the wing sizes are a detailed reflection of an individual's underlying genetics the pattern of backcrossing is also evident in the wing data presented here. Wing lengths of hybrid individuals from Guinea-Bissau were more similar to those of the M form (Fig. 1A) while wing widths of hybrids were more similar to those of the S form (Fig. 1B) although neither comparison was statistically significant (Table 1).

Mosquito wing size is regularly used as a measure of overall body size and hence individual fitness [29]. It is also a phenotype subject to fluctuations dependent upon environmental factors [30]. A recent study in Ghana revealed that wing size, as measured by wing length, fluctuates with season in *A. gambiae* s.s., with those collected in the wet season being larger than those collected in the dry season [31]. In the study presented here, all mosquitoes were collected in the wet season. However, it is unknown whether the difference in wing size between countries, observed in the present study, can be attributed to a genetic difference, developmental plasticity in response to local environmental conditions or a combination of these components. It is also important to note that the larval habitat of the mosquitoes can have a significant impact on final adult size [30], [32]. The data presented in this study may also support the hypothesis that larval habitat segregation [11], [12] and ecological factors [33] are driving divergence between and within the M and S forms.

### Assortative mating

The data presented here support the hypothesis that wing beat frequency, as measured by wing size, confers information about an individual's underlying genetic make-up. Examples of courtship songs exist in other dipteran insects including the *Drosophila* spp. [34] and Phlebotomine sand flies [35]. However, as has been demonstrated in other mosquito species [16], [18], harmonic convergence is the key to mosquito courtship “song”. Yet the two types of acoustic behaviors are hypothesized to act in the same manner by conveying fitness information and mate quality, thus allowing for mate selection, assortative mating and divergent evolution.

Harmonic convergence between male and female *A. gambiae* most likely occurs at very close range in or near the mating swarm. Swarm segregation based on molecular form has been found to be complete [14] or mixed [15] depending on geographic location. The use of harmonic convergence might be more useful in a mixed swarm but further studies are needed to determine if harmonic convergence and wing size differ in areas where segregated molecular form mating swarms occurs. Overall, harmonic convergence may convey sufficient information about local adaptation of a potential mate regardless of its molecular form that it may also have a role in segregated mate swarms.

### Future studies

The samples analyzed in this study represent a relatively small geographic portion of the sympatric distribution of *A. gambiae* s.s. M and S forms in West Africa. Just as the levels of hybridization vary across the distribution [4], [6], [24], [36], [37], the pattern of wing size differentiation may differ which could be determined with a more widespread sampling distribution. In this same regard, the patterns of wing size and shape differentiation may similarly vary. Thus future studies that take advantage of a geometrics-morphometrics approach to wing shape as well as wing size [38] may provide a more detailed examination of patterns of wing differentiation. In addition, analysis of other structures directly related to mating (e.g. male genitalia) combined with homologous genes related to mating behavior from *Drosophila* spp., as has been done in sand flies [39], [40], may provide us with candidate genes under selection and thus potential targets for study and better understanding of reproductive isolation mechanisms and speciation with gene flow.

### Conclusion

Overall, the data presented here support the hypothesis that pre-mating reproductive isolation mechanisms mediated by wing beat frequency, as measured by wing size, allow for assortative mating between the M and S molecular forms of *A. gambiae*. Furthermore, the data suggest that wing morphology may support genetic investigations and patterns of hybridization observed in the field. The wings of *A. gambiae* may bridge the gap between molecular, ecological and organismal studies of this medically important species.

## Materials and Methods

### Mosquitoes

The mosquitoes examined in this study were collected as part of two larger studies examining aspects of population dynamics and malaria infection in *Anopheles gambiae* in west Africa (NIH grants: AI062929 and AI078183). Wings used in the analyses here were salvaged from these specimens, which had already been dissected into three pieces in the field for other analyses. Collections of mosquitoes did not require any specific permits but were made in collaboration with Sekou Traore (Malaria Research Training Center, University of Bamako, Faculte de Medicine de Pharmacie et D'Odonto-Stomatologie, Bamako, Mali) and Amabelia Rodriguez (National Institute of Public Health (INASA), Bissau, Guinea-Bissau). Individual house collections were made with the permission of village authorities and individual residents. Mosquitoes from Mali were collected from the villages of Kela and Selenkenyi in September 2010 (Table 2). Samples from Guinea-Bissau were from the areas of Antula, Prabis, Abu, Bambadinca, Eticoga, Bruce, and Canjufa collected in October and November 2009 (Table 2). Although at a few sites listed in Table 2 from Guinea-Bissau, there appears to be only one or two of the forms collected this represents only high quality specimens and is not due to a lack of sympatric populations of the molecular forms at those sites.

**Table 2:** Locations of the mosquito collection sites and sample sizes of the wings used in the analysis of morphological differentiation in *Anopheles gambiae* s.s.

Country	Site	Latitude	Longitude	M	S	Hybrid
**Mali**	Kela	11.88683	−8.44744	9	8	1
**Mali**	Selenkenyi	11.70000	−8.28330	10	8	0
**Guinea-Bissau**	Canjufa	12.43189	−14.12662	0	1	1
**Guinea-Bissau**	Bambadinca	12.02233	−14.86200	10	0	0
**Guinea-Bissau**	Antula	11.91005	−15.58374	6	24	10
**Guinea-Bissau**	Prabis	11.80066	−15.74332	10	12	6
**Guinea-Bissau**	Abu	11.46144	−15.91411	4	12	6
**Guinea-Bissau**	Bruce	11.22844	−15.87547	0	3	0
**Guinea-Bissau**	Eticoga	11.15879	−16.14269	0	4	0

### Molecular form determination

Due to the complexity of hybridization in Guinea-Bissau [21] molecular form determination was accomplished by a consensus of the commonly used PCR methods [27], [28], direct sequencing (UC DNA Sequencing Facility) and Sequenom® iPLEX SNP genotyping (UC Davis Veterinary Genetics Laboratory) following manufacturer protocols. For samples from Mali the PCR techniques commonly used for specimens from this region were used [27], [28] and matched on all samples.

### Wing mounting and measurement

Wings were removed from mosquitoes, noting left and/or right side of the insect, prior to crushing of the head and thorax for detection of malaria parasites for other studies and had been stored in 100% ethanol. Wings from each individual were mounted on microscope slides with Entellan® Rapid Embedding Agent for Microscopy (Electron Microscopy Services, Hatfield, PA, USA) without coverslips. Each wing was prepared for mounting by briefly placing it in a series of baths consisting of 10% potassium hydroxide, to remove wing scales and allow for clear evaluation of wing venation, followed by distilled water and finally 80% ethanol.

Wings were mounted using an Olympus SZ10 dissecting microscope (Olympus Imaging America, Inc., Center Valley, PA, USA) and imaged with an Olympus BX50 (Olympus Imaging America, Inc., Center Valley, PA, USA) compound microscope at 4× magnification. Images were captured with an Olympus DP71 camera using the DP Controller software (Olympus Imaging America, Inc., Center Valley, PA, USA). The images were assembled into a thin-plate-spline format file using tpsUtil version 1.46 (1) and measurements were made in tpsDig2 version 2.12 (2) using the reference scale embedded in each captured image file. Wing images have been posted to the Open Projects page for Island Ecology on the PopI database hosted by the University of California, Davis: https://grassi2.ucdavis.edu/PopulationData/OpenProjects/IslandEcology/.

Wing length was measured as the distance from the posterior anal cell margin to the tip of radial vein 3 (R3). Wing width was measured as the distance from the subcostal junction with the costa, at the leading edge of the wing, to half way between the junctions of the anterior branches of the cubitus veins CuA1 and CuA2 on the trailing edge of the wing (after the naming convention of (3)). All measurements were conducted by the same individual to reduce confounding effects. Due to the fact that wings were salvaged from specimens already processed for other studies we selected intact wings with enough structure to take measurements of either wing length, wing width or both measurements. Only wings from one side of the animal are required for analysis and after evaluation of the images, those from the right side of the mosquitoes were selected, as there were more intact, good quality wings from this side.

### Statistical Analysis

Analyses of wing length and wing width were conducted separately using the General Linear Model procedure in SPSS 16.0 (4). A two-way analysis of variance model was created for each size measurement consisting of the factors: country of wing origin, molecular form of individual and the interaction between these two factors. The Type III sum of squares procedure was implemented to accommodate for the unbalanced sampling of specimens in the study (4). Due to the fact that only a single hybrid specimen was collected from Mali the number of levels within the factor of molecular form was balanced by removing hybrids from the model (complete model provided in Supporting Information S1). The factor of site within country was evaluated prior to the creation of the two-way ANOVA model and no significant effect was observed (Supporting Information S1). The data met the assumptions of analysis of variance and did not require transformation. Significance was observed at the α = 0.05 level.

## Supporting Information

Supporting Information S1 **The supporting information file contains ANOVA tables for the analysis of hybrid forms and the analysis of potential site effects in Mali and Guinea-Bissau.**
(DOC)
