# Supplementary material for: Morphological Differentiation May Mediate Mate-Choice between Incipient Species of Anopheles gambiae s.s
Source: PLoS One. 2011 Nov 21;6(11):e27920. doi: 10.1371/journal.pone.0027920 (PMC3221689; doi:10.1371/journal.pone.0027920)
Supplement: Supporting Information S1 — The supporting information file contains ANOVA tables for the analysis of hybrid forms and the analysis of potential site effects in Mali and Guinea-Bissau. (DOC) [file pone.0027920.s001.doc]

## Supporting Information S1

**Table S1.** **Analysis of variance including hybrid specimens from Guinea-Bissau.** The inclusion of hybrid data from Guinea-Bissau did not change the ANOVA results for between country and between form comparisons. ^a^indicates that the degrees of freedom were affected by the imbalance created by inclusion of the hybrid form.

| **Factor** | **Mean Square** | **d.f.** | **F** | **p-value** |
| --- | --- | --- | --- | --- |
| **Wing Length** |  |  |  |  |
| **Country** | 2331.398 | 1 | 11.469 | 0.001* |
| **Form** | 245.437 | 2 | 1.207 | 0.303 |
| **Form x Country** | 3.589 | 1^a^ | 0.018 | 0.895 |
| **Error** | 203.285 | 110 |  |  |
| **Wing Width** |  |  |  |  |
| **Country** | 271.825 | 1 | 16.578 | <0.001* |
| **Form** | 19.992 | 2 | 1.219 | 0.299 |
| **Form x Country** | 75.942 | 1^a^ | 4.632 | 0.033* |
| **Error** | 16.396 | 128 |  |  |

*indicates a significant difference at the α = 0.05 level

**Table S2. Mali: ANOVA table for site specific wing length and wing width data.** Analysis of site specific wing data within each country. Sites did not have a significant effect on observed wing length or wing widths within each country so all sites were combined for the overall analyses of wing size.

| **Factor** | **Mean Square** | **d.f.** | **F** | **p-value** |
| --- | --- | --- | --- | --- |
| **Wing Length** |  |  |  |  |
| **Site** | 0.001 | 1 | 0.082 | 0.777 |
| **Form** | 0.004 | 1 | 0.266 | 0.611 |
| **Site x Form** | 0.005 | 1 | 0.315 | 0.580 |
| **Error** | 0.016 | 24 |  |  |
| **Wing Width** |  |  |  |  |
| **Site** | 0.000022 | 1 | 0.012 | 0.912 |
| **Form** | 0.007 | 1 | 3.755 | 0.062 |
| **Site x Form** | 0.002 | 1 | 1.302 | 0.263 |
| **Error** | 0.002 | 30 |  |  |

*indicates a significant difference at the α = 0.05 level

**Table S3. Guinea-Bissau: ANOVA table for site specific wing length and wing width data.** Analysis of site specific wing data within each country. Sites did not have a significant effect on observed wing length or wing widths within each country so all sites were combined for the overall analyses of wing size.

| **Factor** | **Mean Square** | **d.f.** | **F** | **p-value** |
| --- | --- | --- | --- | --- |
| **Wing Length** |  |  |  |  |
| **Site** | 210.839 | 7 | 0.929 | 0.490 |
| **Form** | 227.247 | 2 | 1.001 | 0.788 |
| **Site x Form** | 109.732 | 5 | 0.483 | 0.788 |
| **Error** | 223.986 | 72 |  |  |
| **Wing Width** |  |  |  |  |
| **Site** | 16.245 | 6 | 1.021 | 0.417 |
| **Form** | 2.517 | 2 | 0.158 | 0.854 |
| **Site x Form** | 9.701 | 5 | 0.610 | 0.693 |
| **Error** | 15.914 | 85 |  |  |

*indicates a significant difference at the α = 0.05 level
